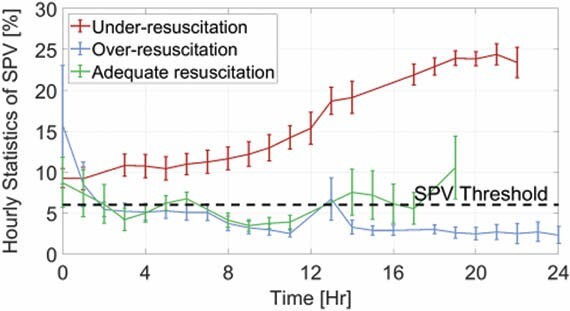# 97 Arterial Waveform Variations as Measures of Resuscitation Adequacy in a Porcine Model of Burn Injury

**DOI:** 10.1093/jbcr/irac012.100

**Published:** 2022-03-23

**Authors:** Ghazal ArabiDarrehDor, Yi-Ming Kao, Mary A Oliver, Adam D Reese, Bonnie C Carney, John W Keyloun, Kevin K Chung, Lauren T Moffatt, Jeffrey W Shupp, Jin-Oh Hahn, David M Burmeister

**Affiliations:** University of Maryland, College Park, College Park, Maryland; University of Maryland, College Park, College Park, Maryland; Burn Center at MedStar Washington Hospital Center, Washington DC, District of Columbia; Burn Center at MedStar Washington Hospital Center, Washington DC, District of Columbia; Medstar Health Research Institute, Washington DC, District of Columbia; Burn Center at MedStar Washington Hospital Center, Washington DC, District of Columbia; Uniformed Services University, Bethesda, Maryland; Burn Center at MedStar Washington Hospital Center, Washington DC, District of Columbia; MedStar Washington Hospital Center, Washington DC, District of Columbia; University of Maryland, College Park, College Park, Maryland; Uniformed Services University, Bethesda, Maryland

## Abstract

**Introduction:**

Optimized fluid resuscitation of burn patients is a clinical care challenge as both under- and over- resuscitation have deleterious consequences. The gold-standard endpoint guiding burn resuscitation is urinary output (UO), which is known to have limited efficacy. We investigated the potential of the dynamic indices of fluid responsiveness derived from arterial blood pressure (BP) waveforms in conveying information about burn resuscitation. In particular, we investigated pulse pressure variation (PPV) and systolic pressure variation (SPV), which have been shown to be valuable in a number of other indications.

**Methods:**

We conducted a retrospective analysis of arterial BP waveform data acquired from six anesthetized and mechanically-ventilated pigs (33±5 kg weight and 40% total burned surface area) which were instrumented for hemodynamic monitoring for 24 hours. The animals were either under-, over-, or adequately-resuscitated (guided by a burn resuscitation decision support system), with two animals in each group. PPV and SPV were calculated on an hourly basis. Fluid responsiveness thresholds of 15% and 6% were used respectively for PPV and SPV, as per literature.

**Results:**

All of the animals experienced an immediate rise in PPV and SPV following the injury (PPV and SPV start from large values as seen in Fig. 1 and Fig. 2). In the under-resuscitated group, PPV and SPV increased above the threshold, reaching maximum values in the last eight hours (PPV: 49.8±20%, SPV: 24.7±3.6%), indicating severe hypovolemia. In the over-resuscitated group, PPV and SPV decreased below the threshold, reaching their minimum in the last eight hours (PPV: 8.7±3.6%, SPV: 4.1±1.9%), indicating major hypervolemia. In the adequately-resuscitated group, PPV and SPV maintained closer to the threshold throughout the duration of the experiment, and at the end, PPV was 15.6±4.2% and SPV was 6.2±2.6%.

**Conclusions:**

Our initial results suggest that PPV and SPV may help distinguish under-, adequately-, and over-resuscitated burn patients, and potentially complement UO in the hemodynamic assessment of the burn injury patients.